# A rapid and reliable method for the determination of *Lactiplantibacillus plantarum* during wine fermentation based on PMA-CELL-qPCR

**DOI:** 10.3389/fmicb.2023.1154768

**Published:** 2023-07-17

**Authors:** Jie Wang, Bo Wei, Zhuojun Chen, Yixin Chen, Songyu Liu, Bolin Zhang, Baoqing Zhu, Dongqing Ye

**Affiliations:** ^1^Beijing Key Laboratory of Forestry Food Processing and Safety, School of Biological Science and Technology, Beijing Forestry University, Beijing, China; ^2^Guangxi Key Laboratory of Fruits and Vegetables Storage-Processing Technology, Guangxi Academy of Agricultural Sciences, Nanning, Guangxi, China

**Keywords:** propidium monoazide, CELL-qPCR, *Lactiplantibacillus plantarum*, real-time monitoring, wine

## Abstract

Real-time monitoring of microbial dynamics during fermentation is essential for wine quality control. This study developed a method that combines the fluorescent dye propidium monoazide (PMA) with CELL-qPCR, which can distinguish between dead and live microbes for *Lactiplantibacillus plantarum*. This method could detect the quantity of microbes efficiently and rapidly without DNA extraction during wine fermentation. The results showed that (1) the PMA-CELL-qPCR enumeration method developed for *L. plantarum* was optimized for PMA treatment concentration, PMA detection sensitivity and multiple conditions of sample pretreatment in wine environment, and the optimized method can accurately quantify 10^4^–10^8^ CFU/mL of the target strain (*L. plantarum*) in multiple matrices; (2) when the concentration of dead bacteria in the system is 10^4^ times higher than the concentration of live bacteria, there is an error of 0.5–1 lg CFU/mL in the detection results. The optimized sample pretreatment method in wine can effectively reduce the inhibitory components in the qPCR reaction system; (3) the optimized PMA-CELL-qPCR method was used to monitor the dynamic changes of *L. plantarum* during the fermentation of Cabernet Sauvignon wine, and the results were consistent with the plate counting method. In conclusion, the live bacteria quantification method developed in this study for PMA-CELL-qPCR in *L. plantarum* wines is accurate in quantification and simple in operation, and can be used as a means to accurately monitor microbial dynamics in wine and other fruit wines.

## 1. Introduction

Lactic Acid Bacteria (LAB) are Gram-positive bacteria, grouped in the phylum Firmicutes, class Bacilli, order *Lactobacillales*. They play many vital roles in food fermentation, such as improving the taste and texture of the food matrix (Costa et al., 2013), enhancing the probiotic effect in the human body (Pereira et al., 2011), and prolonging the shelf life of food (Chwastek et al., 2016). Currently, the main role of LAB in wine is to conduct malolactic fermentation (MLF). This process can increase wine aroma and mouthfeel, improve microbial stability and reduce the acidity of wine (Krieger-Weber et al., 2020; López-Seijas et al., 2020; Brizuela et al., 2021). A growing number of studies support the appreciation that LAB can also significantly, positively, and negatively, contribute to the sensorial profile of wine through many different enzymatic pathways (Bartowsky, 2009). At present, *Oenococcus oeni* has been recommended as a starter due to its high resistance against high alcohol level and low pH environment after the alcoholic fermentation (G-Alegria et al., 2004; Pozo-Bayon et al., 2005; Brizuela et al., 2021). However, with increasing temperatures during growth and harvest, and a consequent rising pH trend for many wines, *Lactiplantibacillus plantarum* have the potential to become a valid alternative to *Oenococcus*, playing an important role in the modifications of wine aroma (Krieger-Weber et al., 2020; López-Seijas et al., 2020; Sun et al., 2020; Wang et al., 2020). Above all, *Lactiplantibacillus* strains, with their fast consumption of malic acid (up to 3 g/L in 2–4 days) and the suppression of the activity of other spontaneous LAB populations, are an ideal starter choice for the winemaker (Du Toit et al., 2011; Krieger-Weber et al., 2020).

The taste, color and aroma of wine are closely related to the growth and metabolism of microorganisms. A variety of enzymes from MLF starters act in the deacidification of wine, and their products directly affect the wine aroma (Albergaria and Arneborg, 2016). Thus, in order to obtain better quality wines without economic loss caused by spoilage microorganism growth, it is necessary to establish a rapid and reliable method on detecting and identifying viable microorganisms in vinification.

Plate counting as a culture-dependent counting method has been widely used to count microorganisms in the winemaking process (Bartle et al., 2021; de Lima et al., 2021). However, its time-consuming process on plating and the lack of capacity on counting slow-growing or viable but non-culturable (VBNC) bacteria could underestimate the density of the microbial population (Roussel et al., 2018). It has been reported that dead microorganisms and the VBNC bacteria could account for more than 60% of the total microbial population during wine fermentation process (Quiros et al., 2009).

Recently, molecular method using microorganism strains DNA has been investigated and developed to replace the culture-dependent counting method (Nocker et al., 2006; Fittipaldi et al., 2012). Real-time quantitative PCR (qPCR) has been applied as a specific culture-independent assay to measure the microbial population in various medium conditions (Li et al., 2021). However, the qPCR technique lacked the ability of differentiating DNA in the dead bacteria from live bacteria. This could create a false amplification of live microbial strains DNA, and further result in an overestimate on the microbial population (Cocolin and Mills, 2003). Propidium monoazide (PMA) has been reported to be introduced to improve the accuracy of the qPCR technology on counting microbial population (Zhang et al., 2020). PMA, as a photosensitive DNA-ligand dye, could penetrate the membranes of dead microbial cells and further cross-link dead cells DNA under intensive light. As a result, the qPCR amplification of DNA from dead microbial cells was inhibited (van Frankenhuyzen et al., 2011; Tantikachornkiat et al., 2016). Meanwhile, live microbial cells possess the intact cell wall structure, which prevents PMA from entering the cell membranes to interact with DNA (Nocker et al., 2006). It has been confirmed that the qPCR inhibitors present in matrix could play a negative role in the DNA denaturation and polymerase action capacity, which resulted in a reduction on qPCR amplification efficiency (Wilson, 1997). Meanwhile, high level of ethanol, polysacharrides and tannins in wine could further interfer the extraction and purification of DNA samples (Tessonniere et al., 2009).

CELL-qPCR method has received much attention on counting the microbial population in wine. This technique eliminates the DNA extraction process and directly utilizes cells as template for the qPCR amplification. This microbial population counting method shortens the reaction time and lowers the reaction cost (Soares-Santos et al., 2017, 2018). To our best knowledge, CELL-qPCR method, coupled with the PMA treatment, has not been studied on microbial kinetics in wine-making process. In the present study, we aimed to develop and optimize a PMA-CELL-qPCR assay to accurately measure the live *L. plantarum* in wine. The established method was further applied to the vinification process to measure the microbial kinetics. The findings from this study could help elucidate the microbial growth mechanisms during wine fermentation and further provide useful insight on quality improvement of wine-making process.

## 2. Materials and methods

### 2.1. Strains and growth media

A lactic acid bacteria strain *L. plantarum* (Lp39, CICC6240) were received from China Center of Industrial Culture Collection (Beijing, China). There *L. plantarum* strains, B3, B4 and SS6 from blueberries, were isolated, identified and stored in our laboratory. The *L. plantarum* strain was cultured in MRS medium (Difco, Maryland, United States) at 37°C for 24 h. The culture was diluted 10 times in a gradient and plated onto the corresponding solid medium. The colony counts in the culture were determined as colony-forming unit (CFU) in triplicate. The absorbance value (OD) was determined at the 600 nm wavelength under a spectrophotometry (Meipuda instrument Co., Ltd., Shanghai, China).

### 2.2. Cell suspension treatments

For microbial strain (*L. plantarum*), four cell suspension samples were prepared, including live microbial cells of the strain without the PMA treatment (control), live microbial cells of the strain with the PMA treatment, dead microbial cells of the strain with the PMA treatment, and mixture of live and dead cells of the strain with the PMA treatment. To verify the efficacy of the PMA treatment on the non-viable cells, a microbial cell suspension was thermally treated at 85°C for 20 min in an autoclave to damage the membranes of all microbial cells. The heat-treated microbial cells were confirmed to be dead by culturing these microbes on the MRS medium.

All cell suspensions were processed as follows prior to qPCR: the cells in the culture medium suspension without the PMA treatment were washed twice using 1 vol ultrapure water. During each washing, the culture was mixed with 1 vol ultrapure water and then centrifuged at 12,000 g (Lab Net International Inc., New Jersey, United States). The cells in the grape must and wine were initially washed with 1 vol ultrapure water, 1 vol 10% TEN buffer (0.1 M Tris–HCl pH 7.5, 0.05 M EDTA, 0.8 M NaCl), and then 1 vol ultrapure water twice. The cell suspension treated with PMA was washed using normal saline, rather than ultrapure water before the PMA treatment.

### 2.3. PMA treatment optimization

A PMA stock solution (20 mM) was prepared by dissolving PMA (Biotum, Hayward, USA) in 20% dimethyl sulfoxide (Sigma-Aldrich, St. Louis, MO, USA). The PMA stock solution was stored at −20°C in the dark. Different aliquots of the PMA solution were incorporated into 1 mL cell suspension to generate different PMA concentrations (0, 5, 10, 25, 50, and 100 μM). Afterwards, the PMA-treated cell suspensions were incubated in the darkness for 20 min under constant agitation at 200 rpm. After the incubation, the cell suspensions were exposed to a 450-nm blue LED light (LED, 5 mm, 3.7 V, 20 mA, 2600 MCD) at a 7 cm distance to the light source for 10 min under a photo-activation system to induce cross-linking of PMA and cell DNA. After the treatment, the cell suspensions were centrifuged at 12,000 g for 30 s and then washed with 1 vol sterile distilled water. The resultant cell pellets were used for the CELL-qPCR assay. Cell suspensions without the PMA treatment were used as the negative control. Each experiment was performed in triplicate.

### 2.4. DNA extraction

The lactic acid bacteria genomic DNA in cell suspension (1 mL) from the culture medium or wine was extracted using the Bacterial DNA Extraction Kit (Beijing Tiangen Biochemical Technology Co. Ltd., Beijing, China). The extraction procedure followed the manufacturer’s instructions.

### 2.5. Primers

Primers (*L.p* - f: 5’-TGATCCTGGCTCAGGACGAA-3′; *L.p* - r: 5’-TGCAAGCACCAATCAATACCA-3′) were used for the quantification of *L. plantarum*. Sequences and characteristics of the primers were listed in Table 1.

**Table 1:** Primer sequences developed for CELL-qPCR and PMA-CELL-qPCR.

	Target gene	Length (bp)	Forward primer (5′ → 3′)	Reverse primer (5′ → 3′)	References
*L. plantarum*	Tal	160	AACATTTCGCGGAACTTGGTT	ATCATCTCTTCGGCCTTGGT	Xiong et al. (2019)
	16S rDNA	341	AGCAGTAGGGAATCTTCCA	CACCGCTACACATGGAG	Zeng et al. (2018)
	16S rDNA	121	ACGCGAAGAACCTTACCAGG	CCCAACATCTCACGACACGA	Kántor et al. (2016)
	recA	68	AGGCGCGGCTGATGTCA	CGCGATTGTCTTGGTTTTGTT	Stevenson et al. (2006)
	16S rDNA	81	TGA TCC TGG CTC AGG ACG AA	TGC AAG CAC CAA TCA ATA CCA	Fiocco et al. (2008)

### 2.6. qPCR

The total volume of the PCR amplification reaction system is 25 μL, and the amplification reaction was carried out using 12.5 μL 2x FastStart Essential DNA Green Master (Roche) qPCR Mix Plus (ROX) (Solis Bio Dyne, Tartu, Estonia) and 1 μL each primer. 2 μL extracted DNA was used for the DNA quantification, whereas 10 μL cell suspension was used for the cell quantification, and the rest is supplemented with sterile water. The qPCR amplification condition was as follows: 95°C for 15 min, followed by 40 cycles at 95°C for 15 s, and then 60°C for 20 s. All the samples were automatically processed for the melting curves analyses of the amplified DNA to determine the reaction specificity. The melting curves were obtained by slow heating from 60°C to 95°C at 0.5°C every 5 s, with continuous fluorescence collection. All the analyses were performed in triplicate in a C100™ Thermal Cycler, FQD-96A Real-Time System (Bio Rad, Richmond, CA, United States). The cycle threshold (Ct) was determined automatically by the instrument after setting the baseline at 100 relative fluorescence units (RFU). The data analysis was carried out with the BioRadFQD-96A Manager Software (version 2.1, Richmond, CA, United States). Negative controls were included.

### 2.7. Standard curves

Standard curves were generated by plotting the Ct values of the qPCR against different concentrations of cells (10^2^ to 10^9^ cells/mL). Standard curves were made for each strain in the culture medium, grape must, and wine in triplicate.

### 2.8. Applying PMA-CELL-qPCR to different wine matrices

Two different origins of Cabernet Sauvignon grapes (Huailai and Pinggu) were destemmed and crushed to obtain grape juice. After activated the wine yeast powder for two generations and reached a final cell concentration of 10^8^ CFU/mL at the logarithmic phase after 12 h of growth, it was used as a fermentation agent. The yeast was inoculated at a rate of 1% into each fermentation flask (approximately 300 mL per flask in 500 mL conical flasks). The initial yeast concentration was 10^6^ CFU/mL. After the completion of alcoholic fermentation, the different wines were filtered, sterilized. *L. plantarum* (LP39) was activated from the bacterial powder for two generations and reached a final cell concentration of 10^9^ CFU/mL at the logarithmic phase after 8 h of growth. It was then inoculated at a rate of 4% into a simulated wine environment for acclimation and subsequent generations. After 8 h of acclimation, it was inoculated into the wine samples at a rate of 1%, resulting in a concentration of 10^7^ CFU/mL.The wines were sampled every 3 days during the fermentation period. The number of *L. plantarum* cells in the wine samples was measured using CELL-qPCR, PMA-CELL-qPCR, and plate counting.

### 2.9. Measurement of physicochemical parameters

The content of reducing sugar in the wine samples was determined using the national standard method for physical and chemical analysis of wine (GB/T15038-2006). This method provides standardized procedures for measuring the reducing sugar content in wine, ensuring consistency and accuracy in the results obtained. By following the guidelines specified in this standard, the reducing sugar content in the wine samples can be quantitatively determined. When the content of reducing sugar in the wine samples is below 4 g/L, it is considered that alcoholic fermentation has ended.

The determination of malic acid content was performed using a Malic Acid Assay Kit (Megazyme). Following the instructions provided with the kit, the diluted samples were added to the corresponding wells of the microplate. The absorbance values were then measured at a wavelength of 340 nm using a microplate reader. Four points were measured for each well (2×2 grid), and the average value (A) was obtained. The malic acid content was calculated using the following formula:

Malic acid content $=\frac{\Delta A\;\mathrm{Sample}}{\Delta A\;\mathrm{Standard}}\times \mathrm{Standard}\times$ Dilution factor.

The dilution factor in the experiment was 10-fold. The malic acid content of the standard solution was 0.15 g/L. ΔA (standard) represents the absorbance difference of the standard solution before and after the reaction, and ΔA (sample) represents the absorbance difference of the sample before and after the reaction.

### 2.10. Applying PMA-CELL-qPCR to wine

To assess the applicability of PMA-CELL-qPCR method across various *Lactiplantibacillus* strains and to investigate whether *L. plantarum* can independently complete malolactic fermentation, fermentation experiments were conducted using four strains of *L. plantarum* (LP39, SS6, B3, B4) and one strain of *O. oeni*. The four *L. plantarum* strains were individually inoculated (designated as LP39, SS6, B3, B4), along with a 1:1 mixture of *L. plantarum* and *O. oeni* (designated as LP39 + *O. oeni*, SS6 + *O. oeni*, B3 + *O. oeni*, B4 + *O. oeni*), at a concentration of 10^7^ CFU/mL for a 19-day malolactic fermentation (MLF) period. The viable bacterial population in the wine during this process was assessed using both plate counting and PMA-CELL-qPCR techniques.

### 2.11. Statistical analysis

All the statistical analyses were performed using version 8.0 of the GraphPad Prism software (San Diego, CA, United States). Student’s t-test with a 5% significance level was used to determine the significant differences among the CELL-qPCR, PMA-CELL-qPCR, and plate counting quantification.

The PMA-CELL-qPCR and CELL-qPCR standard curves were statistically analyzed using one-way ANOVA under Tukey’s multiple comparisons test to determine the effect of the matrix on cell quantification ability. One-way ANOVA under Dunnett’s multiple comparisons test on the PMA-CELL-qPCR and CELL-qPCR standard curves was applied to determine the specificity of the PMA-CELL-qPCR assay. The statistical degree of significance was set at a value of *p* of <0.05.

## 3. Results and discussion

### 3.1. Determination of the optimal PMA concentration

It has been reported that PMA, a membrane-impermeable dye, can selectively penetrate compromised cell membranes of dead cells, leading to the degradation of a portion of the genomic DNA (Nocker et al., 2006; Yáñez et al., 2011). The concentration of PMA has also been found to determine the efficacy of removing dead cell DNA in mixed bacterial cultures containing both live and dead cells, which can impact the accuracy of live cell quantification using the PMA-qPCR method (Nocker et al., 2006). Therefore, optimizing the PMA concentration for different microorganisms is necessary to ensure reliable quantification of microbial populations using the PMA-qPCR technique (Fittipaldi et al., 2012; Reyneke et al., 2017; Berninger et al., 2018).

**Figure 1:**
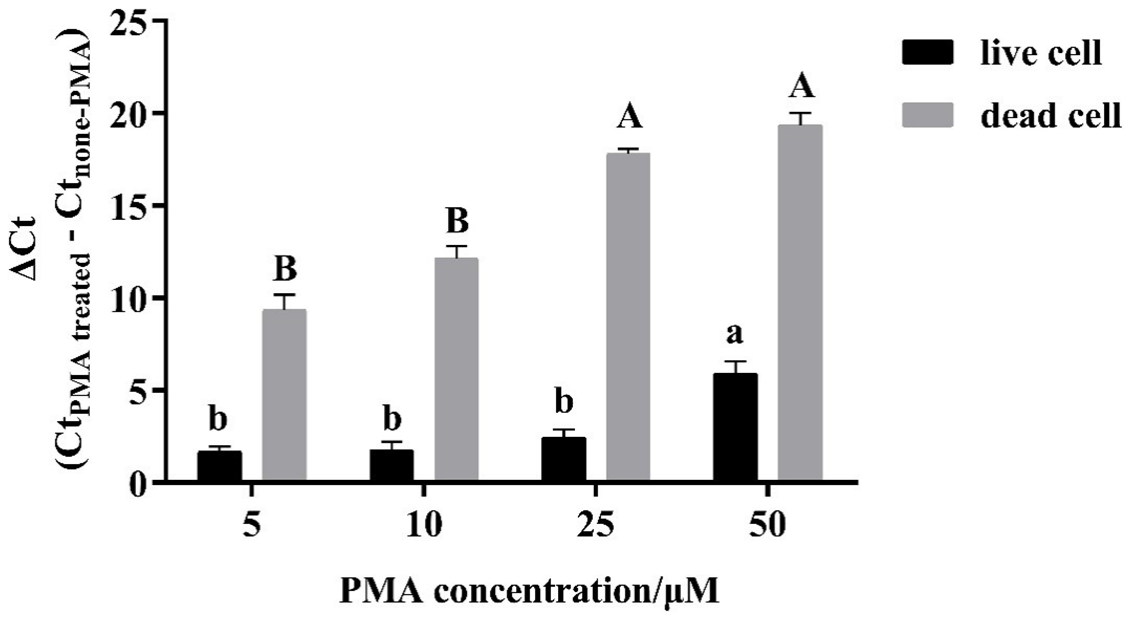
Effect of PMA concentration on PMA-CELL-qPCR signals of live/dead cells of *L. plantarum*. Signal reductions (ΔCt) were derived by subtracting Ct values of non-PMA treated cells from the Ct values of PMA treated cells.

In the present study, the live and dead *L. plantarum* cells with the cell concentration at 10^8^ CFU/mL were separately treated with different PMA concentrations and then counted using the CELL-qPCR assay. Their cycle thresholds (Ct) were further compared to those without the PMA treatment to determine the optimal ΔCt value. It was found that the PMA concentration increase resulted in a significant increase on the ΔCt value in the dead *L. plantarum* cells (Figure 1). The ΔCt value appeared to be 17.80 in the dead *L. plantarum* cells treated with the 25 μM PMA compared to the cells without the PMA treatment. The ΔCt value at the 25 μM PMA showed the significant difference in comparison of that with the PMA concentration at 5 μM and 10 μM (*p* < 0.05). Meanwhile, the dead *L. plantarum* cells treated with the 25 μM PMA concentration were not found to exhibit the fluorescence signal. It should be noted that no significant difference (*p* > 0.05) on the ΔCt value was found in the dead cells treated with the 25 μM and 50 μM PMA concentration. The present study also investigated the effect of different PMA concentration on the DNA amplification of the live *L. plantarum* cells. It was found that no significant differences on the ΔCt value were observed in the live cells when the *L. plantarum* cells were treated with the PMA concentration below 25 μM (p > 0.05). However, the ΔCt value appeared to significantly increase (p < 0.05) when the 50 μM PMA concentration was applied to the live *L. plantarum* cells. These indicated that low PMA concentration (5 μM and 10 μM) could not completely bind with DNA in the dead *L. plantarum* cells, whereas a better interaction occurred between the dead *L. plantarum* cell DNA and PMA under the high PMA concentration treatment (25 μM and 50 μM). However, the high PMA concentration treatment could induce a false determination on the live *L. plantarum* cells counting.

It was found that high PMA concentration resulted in a complete inhibition of the dead cells through interacting with their DNA. However, the excess amount of the PMA molecules induced a reduction on the DNA amplification in the live cells under the CELL-qPCR assay. The mechanism behind this observation has not been elucidated. The similar observations have been reported. For instance, a slight cytotoxic effect was reported to be found in live *Listeria* cells treated with the 50 μM PMA concentration (Pan and Breidt, 2007). Similarly, it was reported that high PMA concentration treatment resulted in a significant reduction on the live *Lactobacillus bulgaricus* cells counting (Shao et al., 2016). The present study indicated that the optimal PMA concentration for *L. plantarum* was at 25 μM. At such PMA concentration, the dead cells appeared to be significantly eliminated from the cell population counting and no negative effect on the live cell DNA amplification was observed.

### 3.2. Standard curve in pure medium environment

To validate the CELL-qPCR and PMA-CELL-qPCR methods on counting the cell population, the amplification using these methods were compared with the DNA amplification using qPCR under the same cell suspension. Table 2 shows the correlation between the Ct value and the cell concentration. The qPCR assay showed that a linear relation was established between the Ct value and the cell concentration over a range of 6 magnitude orders (10^4^ CFU/mL to 10^9^ CFU/mL), with the limit of detection at 10^4^ CFU/mL and the correlation coefficient above 0.98. It should be worth noting that both CELL-qPCR and PMA-CELL-qPCR assays exhibited a significant difference on the detection sensitivity and limit of detection compared to the qPCR assay. A linear standard curve was found in the *L. plantarum* cells under the cell concentration of 10^4^ CFU/mL to 10^8^ CFU/mL using the CELL-qPCR and PMA-CELL-qPCR quantification. It should be noted that the standard curve of the cells using the qPCR method exhibited a wider cell concentration range than the CELL-qPCR and PMA-CELL-qPCR method. The similar results were also reported in other studies. For example, the standard curve of *B. bruxellensis*, *O. oeni* and *A. acet* using qPCR method exhibited a greater cell concentration range than CELL-qPCR assay (Soares-Santos et al., 2017). In the present study, all the amplification methods showed the good amplification efficiencies (0.8 to 1.2) with qPCR slightly outperforming the other two assays in the cells (Table 2). However, CELL-qPCR, compared to qPCR, significantly reduced the amplification process. Meanwhile, its correlation coefficient, amplification efficiency and detection limit were all comparable with those under qPCR method. Therefore, CELL-qPCR could be used as an alternative to qPCR when it comes to the quantitative analysis of the strains. Additionally, the standard curves of both cell strains generated using the PMA-CELL-qPCR method were similar as those under the CELL-qPCR assay in terms of the cell concentration range, detection limit and correlation coefficient. These indicated that the PMA-CELL-qPCR exhibited a good reproductivity and this method could become a validated method on quantifying the viable bacterial populations.

**Table 2:** Correlation coefficients, slopes, and efficiencies of standard curves (log of plate count value vs. cycle threshold) constructed by qPCR, CELL-qPCR, PMA-CELL-qPCR using species specific primers in purity culture and wine.

Method	Species	Matrix	Equation of linear regression	*R*^2^	Efficiency	Limit of detection (CFU/mL)
qPCR	*L. plantarum*	Purity culture	y = −3.716x + 46.48	0.9888	0.8399	10^4^–10^9^
CELL-qPCR	*L. plantarum*	Purity culture	y = −3.329x + 44.03	0.9859	0.9971	10^4^–10^8^
		Huailai	y = −3.363x + 46.68	0.9933	0.9996	10^4^–10^8^
		Pinggu	y = −3.035x + 45.35	0.9818	1.1354	10^4^–10^8^
PMA-CELL-qPCR	*L. plantarum*	Purity culture	y = −3.906x + 44.23	0.9944	0.8030	10^4^–10^8^
		Huailai	y = −3.462x + 47.65	0.9820	0.9446	10^4^–10^8^
		Pinggu	y = −3.278x + 46.58	0.9880	1.0187	10^4^–10^8^

### 3.3. Effect of dead bacteria on PMA treatment efficiency

The PMA-CELL-qPCR method in the present study was proposed to monitor the viable *L. plantarum* population in wine fermentation process. Therefore, it is necessary to evaluate if the dead microbial cells during the vinification process could affect the accuracy of the method. It was found that *L. plantarum* cells could be accurately quantified using the standard curve through the PMA-CELL-qPCR assay under a cell concentration range of 10^4^ CFU/mL to 10^8^ CFU/mL (Table 2). To evaluate how dead bacterial cells affect the PMA treatment efficiency, the dead *L. plantarum* cells with three different concentration (10^4^ CFU/mL, 10^6^ CFU/mL, and 10^8^ CFU/mL) were mixed with the live *L. plantarum* cells (10^4^ CFU/mL or 10^8^ CFU/mL) under a 1:1 volumetric ratio, the Ct value of the mixed cells treated with and without PMA were compared.

**Figure 2:**
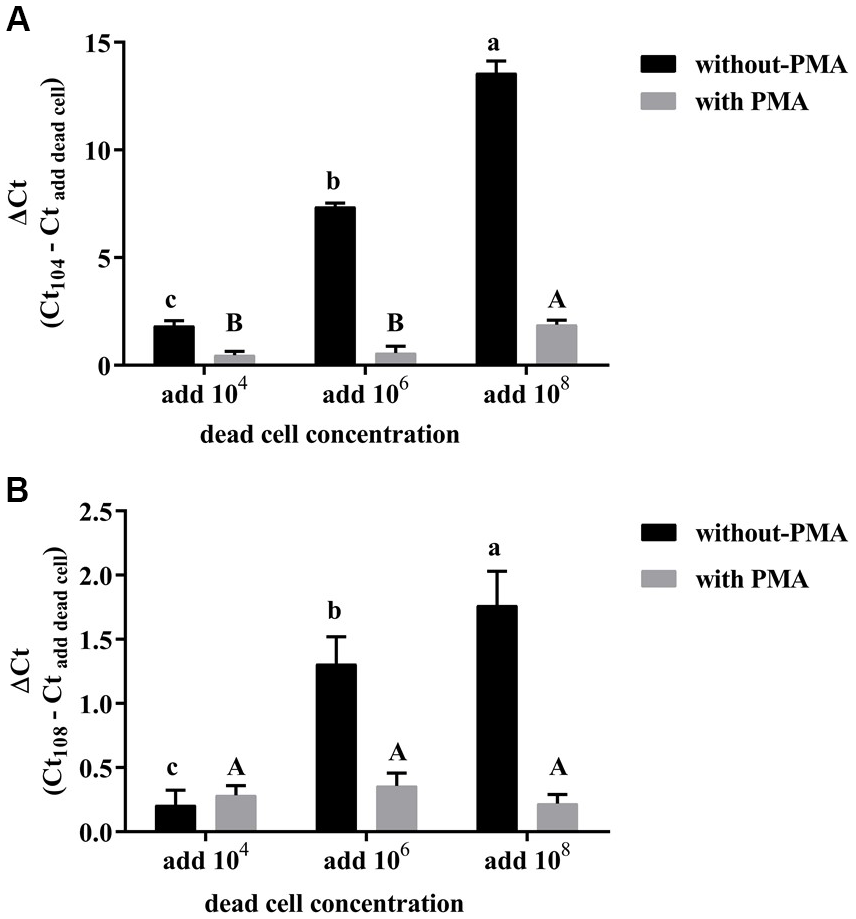
Effects of different concentrations of dead cells on the detection of 10^4^ CFU/mL **(A)**, 10^8^ CFU/mL **(B)** *L. plantarum* by PMA-CELL-qPCR.

It was found that the increase of the dead cells concentration in the live cells resulted in a significant increase on the Ct value when the microbial population was quantified using CELL-qPCR (Figure 2). This indicated that the CELL-qPCR method could not differentiate the dead cells from the live cells, and the microbial population was quantified in combination of the dead and live cells. The PMA treatment resulted in a significant improvement on the live cells quantification under CELL-qPCR. For example, the *L. plantarum* live cells (10^4^ CFU/mL) mixed with 10^4^ CFU/mL and 10^6^ CFU/mL dead cells exhibited the similar (*p* > 0.05) Ct value compared to the live cells without the dead cell mixing (Figure 2A). It should be noted that the ΔCt value appeared to significantly increase (*p* < 0.05) in the live cells (10^4^ CFU/mL) mixed with 10^8^ CFU/mL dead cells. This indicated that the excessive amounts of dead cells could inhibit the PMA molecules to enter the dead cells, lowering the DNA interaction efficiency (Zhao et al., 2019). The PMA-CELL-qPCR technique could effectively inhibit the amplification of the dead cells when the live cells population was higher than or equal to the dead microorganism population. Our result was consistent with a published report where PMA-qPCR appeared to accurately determine the population of viable *Staphylococcus aureus* cells when the live to dead cell ratio was below 1:1000 (Takahashi et al., 2018). Regarding the *L. plantarum* live cells at the concentration of 10^8^ CFU/mL, the incorporation of the dead cells at these three concentration levels did not alter the ΔCt value (*p* > 0.05) (Figure 2B). These indicated that the PMA-CELL-qPCR quantitative analysis could achieve a more reliable result on determination of the viable microbial population in a high viable cells condition, and the false noise produced from the dead cells could be effectively eliminated by PMA. It has been reported that PMA-qPCR accurately quantified the viable cells from the mixture of the dead and live cells conditions, and it turned out that PMA played an essential role on the differentiation of live cells from the dead cells (Zhang et al., 2015). It should be worth noting that the present study illustrated that the PMA-CELL-qPCR assay could provide a detection error below 0.5 on the quantification of the viable cells when the dead-to-live cells ratio was equal or below 10^4^. When the dead-to-live cells ratio was below 10^2^, the PMA-CELL-qPCR method could eliminate the dead cells false determination noise and provide an accurate and reliable quantification of the viable cell population.

### 3.4. Grape must and wine standard curves

It has been reported that tannins, polysaccharides, polyphenols and ethanol in grape must and wine played critical roles in inhibiting the PCR amplification (Demeke and Jenkins, 2010). Further studies have also suggested that red wine and white wine with high level of polyphenols could result in a stronger inhibition on the PCR amplification (Tofalo et al., 2012). Therefore, two types of wine matrices derived from two regions, including Pinggu and Huailai, were used in the present study. The microorganism strain (*L. plantarum*) was cultured in the wine matrix to the microbial population at 10^9^ CFU/mL. The cell suspension was then diluted using the same wine matrix to the concentration of 10^1^ CFU/mL to 10^9^ CFU/mL. It has been reported that a water-soluble polymer, PVP, could interact with polyphenols and it has been widely used to eliminate the effect of polyphenols and other components in wine on the DNA amplification (Castillo-Sánchez et al., 2008; Jara et al., 2008; Tessonniere et al., 2009; Torija et al., 2010). In the present study, the cell suspensions were washed using the TEN buffer supplemented with PVP.

Regarding the *L. plantarum* cells (Table 2), the standard curves in both wine matrices generated using both PMA-CELL-qPCR and CELL-qPCR exhibited the good parallelism (R^2^ > 0.98). The amplification efficiency of *L. plantarum* cells in the Pinggu wine matrix using the PMA-CELL-qPCR technique appeared to be 1.018, whereas the CELL-qPCR amplification efficiency on the *L. plantarum* cells from the Pinggu matrix was found to be 1.135. In the Huailai grape wine matrix, the amplification efficiency on the *L. plantarum* cells using both assays turned out to be quite similar. These results demonstrated that the PMA pretreatment in wine environment did not affect the amplification accuracy of *L. plantarum*. It should be worth noting that both CELL-qPCR and PMA-CELL-qPCR exhibited an accurate quantification of the *L. plantarum* population in the wine matrices with the microbial concentration rang of 10^4^ CFU/mL to 10^8^ CFU/mL. These results were in accordance with a published study where the *L. plantarum* cell population could be quantified in red wine under the *L. plantarum* microbial concentration of 10^4^ CFU/mL to 10^8^ CFU/mL (Soares-Santos et al., 2017). It has been reported that qPCR assay could quantify *L. plantarum* microorganisms with a microbial concentration range of 10^3^ CFU/mL to 10^8^ CFU/mL (Neeley et al., 2005; Cangelosi et al., 2010). During the malolactic fermentation in wine, *L. plantarum*, compared to the traditional malolactic starter (*O. oeni*), has been reported to possess higher reproduction rate with a low nutrient requirement (Brizuela et al., 2017). Meanwhile, *L. plantarum* has been accepted to possess strong resistance on high alcohol environment, and it has been reported that the viable *L. plantarum* population after the course of the malolactic fermentation in wine could still remained around 10^4^ CFU/mL (Bravo-Ferrada et al., 2014).

### 3.5. Effect of wine matrices on the PMA-CELL-qPCR method

**Figure 3:**
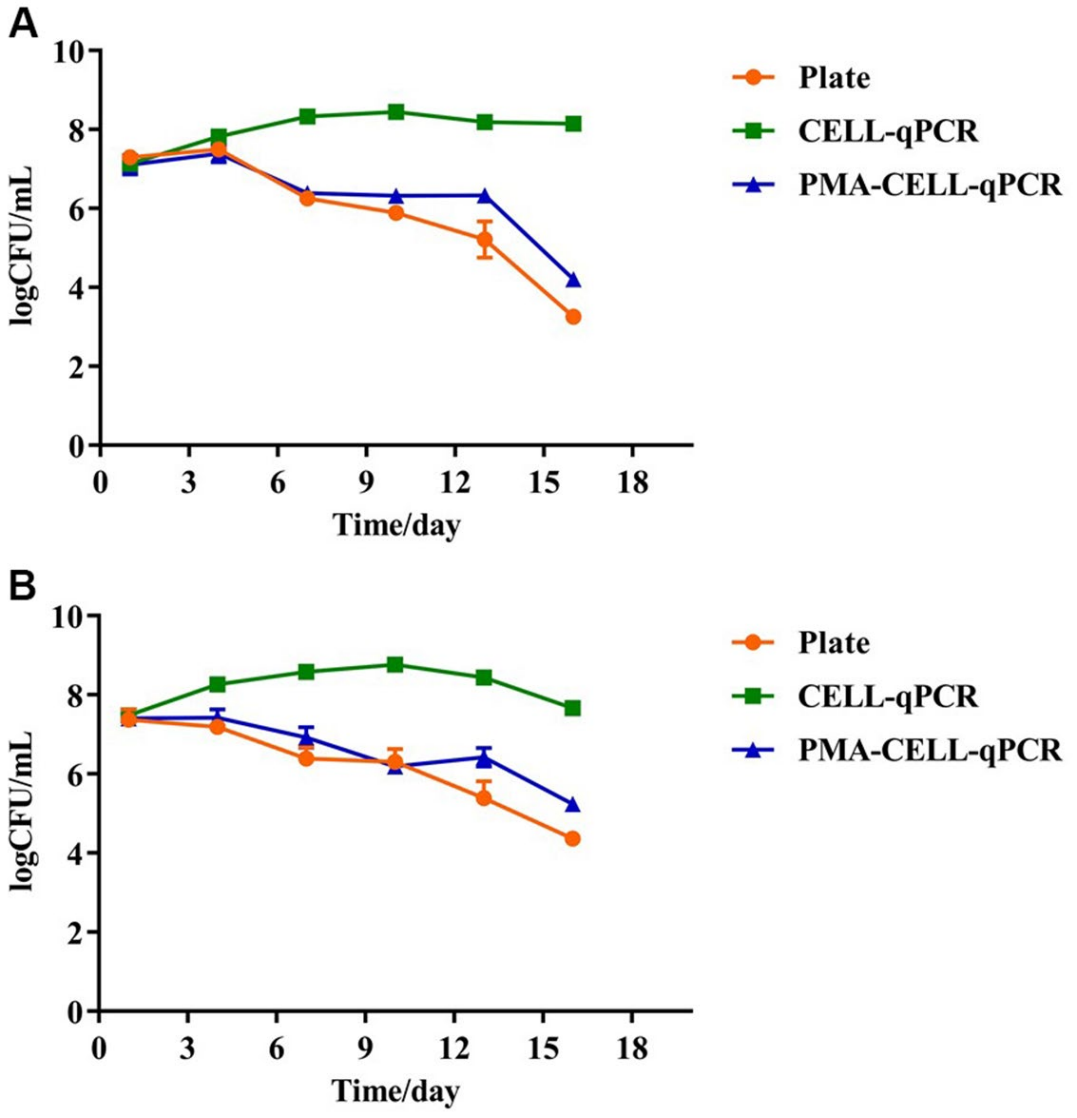
Quantitative analysis of four strains of *L. plantarum* during wine brewing as determined by plate, CELL-qPCR and PMA-CELL-qPCR. Pinggu wine **(A)**, Huailai wine **(B)**.

To further determine the effect of the wine matrix on the PMA-CELL-qPCR method, two types of sterile wines (without yeast and LAB) from Pinggu and Huailai that had completed alcoholic fermentation (the reducing sugar content was less than 4 g/L) were further selected for this study, and the PMA-CELL-qPCR method was used to monitor changes in the bacterial load of *L. plantarum* during malolactic fermentation of the wines. The initial malic acid content of the Pinggu and Huailai wines were 4.33 ± 0.54 g/L and 3.97 ± 0.34 g/L, the physicochemical parameters information were shown in Supplementary Table S1. The wine was inoculated with *L. plantarum* (LP39) at the concentration of 10^7^ CFU/mL to initiate the malolactic fermentation (Figure 3). The whole vinification process lasted 16 days. The viable microbial population in both wine samples were determined using the plate counting, CELL-qPCR and PMA-CELL-qPCR methods, the detailed fermentation monitoring of the strains were shown in Table 3 and Figure 3.

**Table 3:** Quantitative analysis of four strains of *L. plantarum* during Pinggu and Huailai wine brewing as determined by plate, CELL-qPCR and PMA-CELL-qPCR.

	Time	Plate	CELL-qPCR	PMA-CELL-qPCR	*p value*
Pinggu	1 day	7.37 ± 0.25	7.48 ± 0.12	7.77 ± 0.65	NS
	4 day	7.19 ± 0.10 b	8.26 ± 0.13 a	7.59 ± 0.32 b	**
	7 day	6.39 ± 0.28 b	8.58 ± 0.05 a	6.42 ± 0.21 b	**
	10 day	5.64 ± 0.30 b	8.76 ± 0.04 a	5.85 ± 0.39 b	**
	13 day	5.38 ± 0.43 c	7.43 ± 0.11 a	6.42 ± 0.24 b	**
	16 day	4.39 ± 0.34 c	7.26 ± 0.07 a	5.20 ± 0.21 b	**
Huailai	1 day	7.30 ± 0.16	7.63 ± 0.43	7.10 ± 0.25	NS
	4 day	7.49 ± 0.17 b	7.94 ± 0.07 a	7.38 ± 0.22 b	*
	7 day	6.25 ± 0.14 b	8.33 ± 0.09 a	6.38 ± 0.08 b	**
	10 day	5.88 ± 0.15 c	8.45 ± 0.06 a	6.32 ± 0.05 b	**
	13 day	5.21 ± 0.46 c	7.19 ± 0.09 a	6.33 ± 0.07 b	**
	16 day	3.26 ± 0.20 c	7.15 ± 0.06 a	4.21 ± 0.08 b	**

Groups with different letters are significantly different, where “NS” indicates no significant difference between groups (*p* > 0.05). *Indicates significant difference between groups (0.01 < *p* < 0.05), **Indicates highly significant differences between groups (*p* < 0.01).

In the Pinggu wine (Figure 3A), the beginning of fermentation (the 0^th^ – 4^th^ days), there were sufficient nutrients in the wine and the stable growth of *L. plantarum* remained at 10^7^ CFU/mL. There was no significant difference between the results of coated plate, CELL-qPCR and PMA-CELL-qPCR counts at this stage and there was a high concentration of live bacteria in the fermentation system. However, in the middle of malolactic fermentation (the 4th – 10th days), the concentration of viable *L. plantarum* decreased significantly, dropping to 10^5^ CFU/mL on the 10th day, and this decrease in cell count may be correlated with the ethanol content of the wine. It should be noted that during malolactic fermentation, *L. plantarum* possesses rapid growth and metabolism in the wine, with counts using the CELL-qPCR method being 2 log CFU/mL higher on day 7 of fermentation compared to the PMA-CELL-qPCR and plate count methods, a change that further increased to 3 lg CFU/mL at the end of the fermentation process (the 13th day) (malic acid content is 0.18 ± 0.06 g/L). The viability of *L. plantarum* declined continuously for some time after the end of malolactic fermentation.

In the Huailai wine (Figure 3B), there was a steady decline in the viability of *L. plantarum* throughout the malolactic fermentation and, in common with the Pinggu wine, there were no significant differences between the PMA-CELL-qPCR method and the plate count method counts throughout the fermentation process, except on the 13^th^ day. In contrast to the Pinggu wines, counts by the CELL-qPCR method were almost 1 log CFU/mL higher on the 4^th^ day of fermentation compared to the PMA-CELL-qPCR and plate counting methods, and this variation increased with the duration of fermentation, increasing to 3 lg CFU/mL at the end of the fermentation process (the 13th day) (malic acid content is 0.06 ± 0.02 g/L), and at the end of fermentation the counts by the CELL-qPCR method decreased to 7 log CFU/mL. This indicated that different wine conditions could play different roles in the degradation pace of dead microorganisms as well as DNA released from the dead microorganisms.

Since both wine samples contained massive amounts of the dead microbes and the exposed DNA molecules released from the dead microbial cells after the vinification process, the CELL-qPCR method indicated the dead-to-viable cells ratio to be 10^4^. However, the cells population counting error was only found to be 1 lg CFU/mL between the PMA-CELL-qPCR and plate counting assays. It has been reported that the microbial population of *S. cerevisiae* cells in Majiapo wine using qPCR was 10 times different as that determined using the plate counting method (Andorra et al., 2010). The microbial cells counting results determined by the PMA-CELL-qPCR method appeared to be quite consistent with those measured using the plate counting assay in these wine samples during the whole vinification process. This demonstrated that PMA-CELL-qPCR could provide accurate and reliable determination on the viable microorganisms in wine. More importantly, this technique could directly detect and quantify the viable microbial population in wine regardless of the microbial level.

### 3.6. Application of PMA-CELL-qPCR to wines

**Figure 4:**
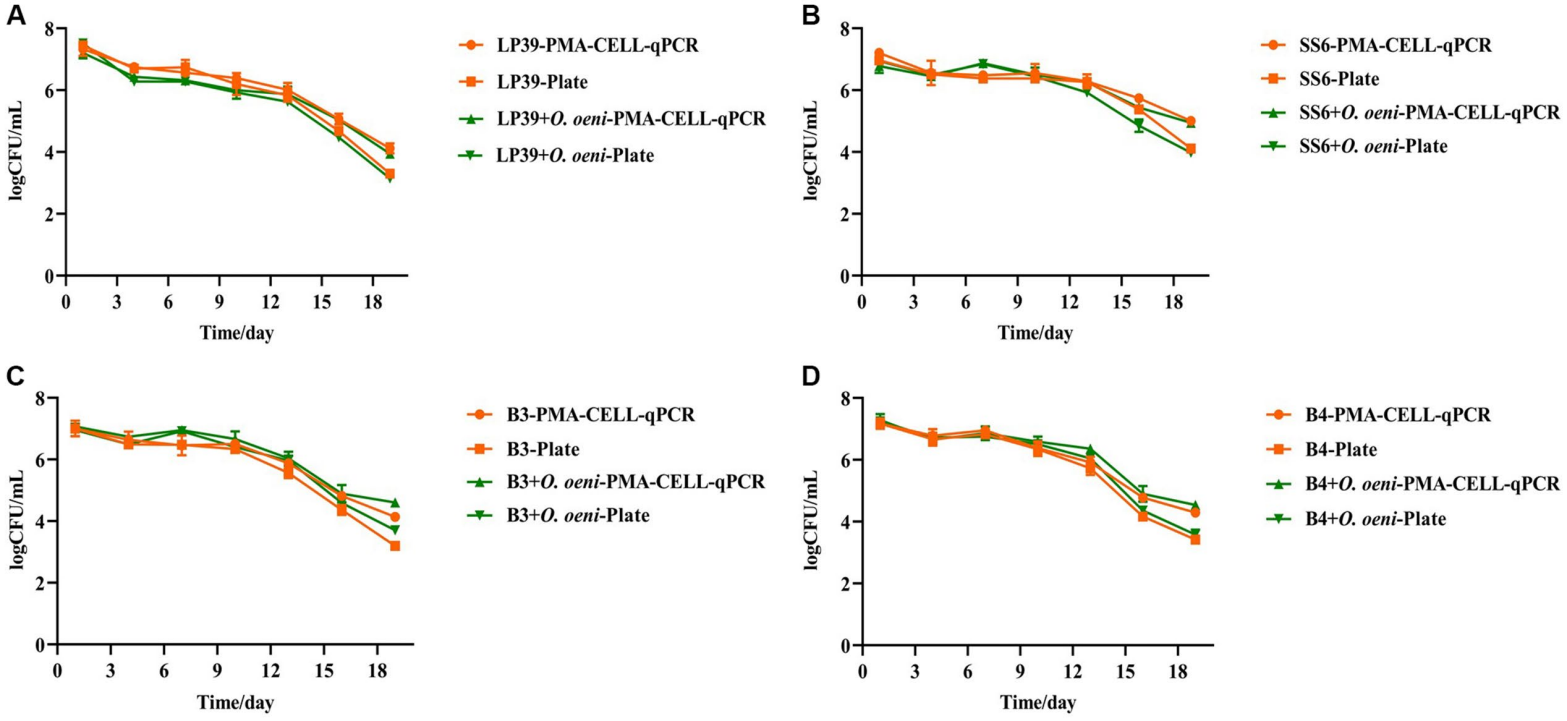
Quantitative analysis of *L. plantarum* during wine brewing as determined by plate and PMA-CELL-qPCR. LP39 **(A)**, SS6 **(B)**, B3 **(C)**, B4 **(D)**.

To further assess the applicability of the PMA-CELL-qPCR method, individual fermentations were conducted using four strains of *L. plantarum* (LP39, SS6, B3, B4). Additionally, co-fermentations were performed by combining these four *L. plantarum* strains with *O. oeni* at a 1:1 ratio (LP39 + *O. oeni*, SS6 + *O. oeni*, B3 + *O. oeni*, B4 + *O. oeni*). The objective was to investigate the quantitative performance of the PMA-CELL-qPCR method in different scenarios and compare it with plate counting results. The quantified results of the different strains throughout the entire fermentation process are presented in Table 4 and Figure 4.

**Table 4:** Quantitative analysis of *L. plantarum* (LP39, LP39 + *O.oeni*; SS6, SS6 + *O.oeni*; B3, B3 + *O.oeni*; B4, B4 + *O.oeni*) during wine brewing as determined by plate and PMA-CELL-qPCR.

Strain	Time	Plate	PMA	*O.oeni*-Plate	*O.oeni*--PMA	*p value*
LP39	1 day	7.44 ± 0.06	7.46 ± 0.38	7.51 ± 0.09	7.10 ± 0.13	NS
	4 day	6.30 ± 0.04	6.54 ± 0.23	6.28 ± 0.04	6.43 ± 0.15	NS
	7 day	6.74 ± 0.24 a	6.56 ± 0.06 ab	6.28 ± 0.04 c	6.31 ± 0.16 bc	*
	10 day	6.20 ± 0.35	6.39 ± 0.14	5.92 ± 0.21	6.00 ± 0.13	NS
	13 day	5.84 ± 0.21	6.01 ± 0.23	5.63 ± 0.13	5.87 ± 0.25	NS
	16 day	5.79 ± 0.10 b	5.93 ± 0.29 b	5.60 ± 0.15 b	6.54 ± 0.38 a	*
	19 day	5.33 ± 0.12 b	5.76 ± 0.22 a	5.15 ± 0.03 b	5.68 ± 0.15 a	**
SS6	1 day	6.96 ± 0.12 ab	7.21 ± 0.13 a	6.94 ± 0.07 ab	6.77 ± 0.22 b	*
	4 day	6.51 ± 0.15	6.55 ± 0.40	6.49 ± 0.11	6.46 ± 0.09	NS
	7 day	6.38 ± 0.13 b	6.47 ± 0.12 b	6.98 ± 0.16 a	7.03 ± 0.21 a	**
	10 day	6.38 ± 0.08	6.55 ± 0.30	6.44 ± 0.03	6.50 ± 0.24	NS
	13 day	6.26 ± 0.11 a	6.28 ± 0.23 a	5.46 ± 0.41 b	5.96 ± 0.09 a	*
	16 day	5.64 ± 0.06 b	5.62 ± 0.34 b	5.66 ± 0.18 b	6.70 ± 0.23 a	**
	19 day	5.64 ± 0.14	5.91 ± 0.20	5.77 ± 0.07	6.13 ± 0.40	NS
B3	1 day	7.00 ± 0.25	7.00 ± 0.15	6.96 ± 0.20	7.07 ± 0.06	NS
	4 day	6.49 ± 0.11	6.64 ± 0.27	6.49 ± 0.12	6.74 ± 0.02	NS
	7 day	6.47 ± 0.13 b	6.46 ± 0.32 b	6.91 ± 0.11 a	7.16 ± 0.21 a	**
	10 day	6.34 ± 0.09	6.50 ± 0.15	6.41 ± 0.07	6.66 ± 0.25	NS
	13 day	5.68 ± 0.14 b	5.74 ± 0.08 b	5.97 ± 0.16 b	6.71 ± 0.31 a	**
	16 day	5.73 ± 0.04 b	5.32 ± 0.16 c	5.89 ± 0.07 b	6.56 ± 0.32 a	**
	19 day	5.79 ± 0.05	5.58 ± 0.21	5.68 ± 0.14	5.81 ± 0.11	NS
B4	1 day	7.17 ± 0.13	7.17 ± 0.17	7.24 ± 0.14	7.28 ± 0.19	NS
	4 day	6.66 ± 0.21	6.78 ± 0.21	6.71 ± 0.01	6.65 ± 0.18	NS
	7 day	6.83 ± 0.14	6.95 ± 0.14	6.75 ± 0.10	6.86 ± 0.23	NS
	10 day	6.08 ± 0.04 b	6.38 ± 0.22 ab	6.59 ± 0.10 a	6.42 ± 0.27 a	*
	13 day	5.72 ± 0.22 b	5.92 ± 0.18 b	6.29 ± 0.03 a	6.42 ± 0.15 a	**
	16 day	5.86 ± 0.09 b	6.28 ± 0.05 b	6.14 ± 0.25 b	7.04 ± 0.45 a	**
	19 day	5.74 ± 0.26	6.09 ± 0.30	5.90 ± 0.05	6.15 ± 0.08	NS

Groups with different letters are significantly different, where “NS” indicates no significant difference between groups (*p* > 0.05). *Indicates significant difference between groups (0.01 < *p* < 0.05), **Indicates highly significant differences between groups (*p* < 0.01).

As can be seen from Figure 4 and Table 4, the overall live counts of the four strains of *L. plantarum* in the wines maintained a steady decline throughout the fermentation process, with a significant decrease in the live counts of *L. plantarum* in all wine samples at the beginning of fermentation (the 0th – 4th days) due to the high alcoholic strength and low pH environment of the wines, reaching a live concentration of 10^6^ CFU/mL on the 4^th^ day of fermentation, with plate counts not significantly different from The results of the PMA-CELL-qPCR method. In the middle of fermentation (the 4th – 10th days), the viability of all four *L. plantarum* strains remained stable at 106 CFU/mL, and the PMA-CELL-qPCR counting method was highly consistent with the plate count results (Figure 4A); in the SS6 and B3 fermented wines, the *L. plantarum* counts were consistently lower in the single-strain fermented samples than in the mixed-strain fermented samples (Figures 4B,C); and in the B4 fermented wines, the inoculation method had no significant effect on the *L. plantarum* counts (Figure 4D). During the later stages of fermentation (the 10th – 19th days), the four strains of *L. plantarum* decreased significantly, probably due to insufficient nutrients during the later stages of fermentation. For the different strains, in wines fermented with LP39 and SS6, *L. plantarum* viability was consistently higher in single-strain fermented samples than in mixed-strain fermented samples (Figures 4A,B); in wines fermented with B3 and B4, *L. plantarum* viability was consistently lower in single-strain fermented samples than in mixed-strain fermented samples (Figures 4C,D), they showed two different growth trends. From the 13^th^ day of fermentation onwards, there was a significant difference between the plate count and the PMA-CELL-qPCR method, with the difference in bacterial load between 0.5 and 1 log CFU/mL. On the one hand, may be due to the presence of strains in the VBNC state in the amount of bacteria detected by the PMA-CELL-qPCR method, while strains in the VBNC state (Zhao et al., 2017), although not detectable by conventional plate counts, are still metabolically active. On the other hand, at the end of fermentation, as the amount of live bacteria was kept at 3–4 log CFU/mL, while the dead bacteria in the system had already reached 8–9 log CFU/mL, the amount of dead bacteria was much larger than the amount of live bacteria by 10^4^ times, which would make the method somewhat inaccurate.

**Figure 5:**
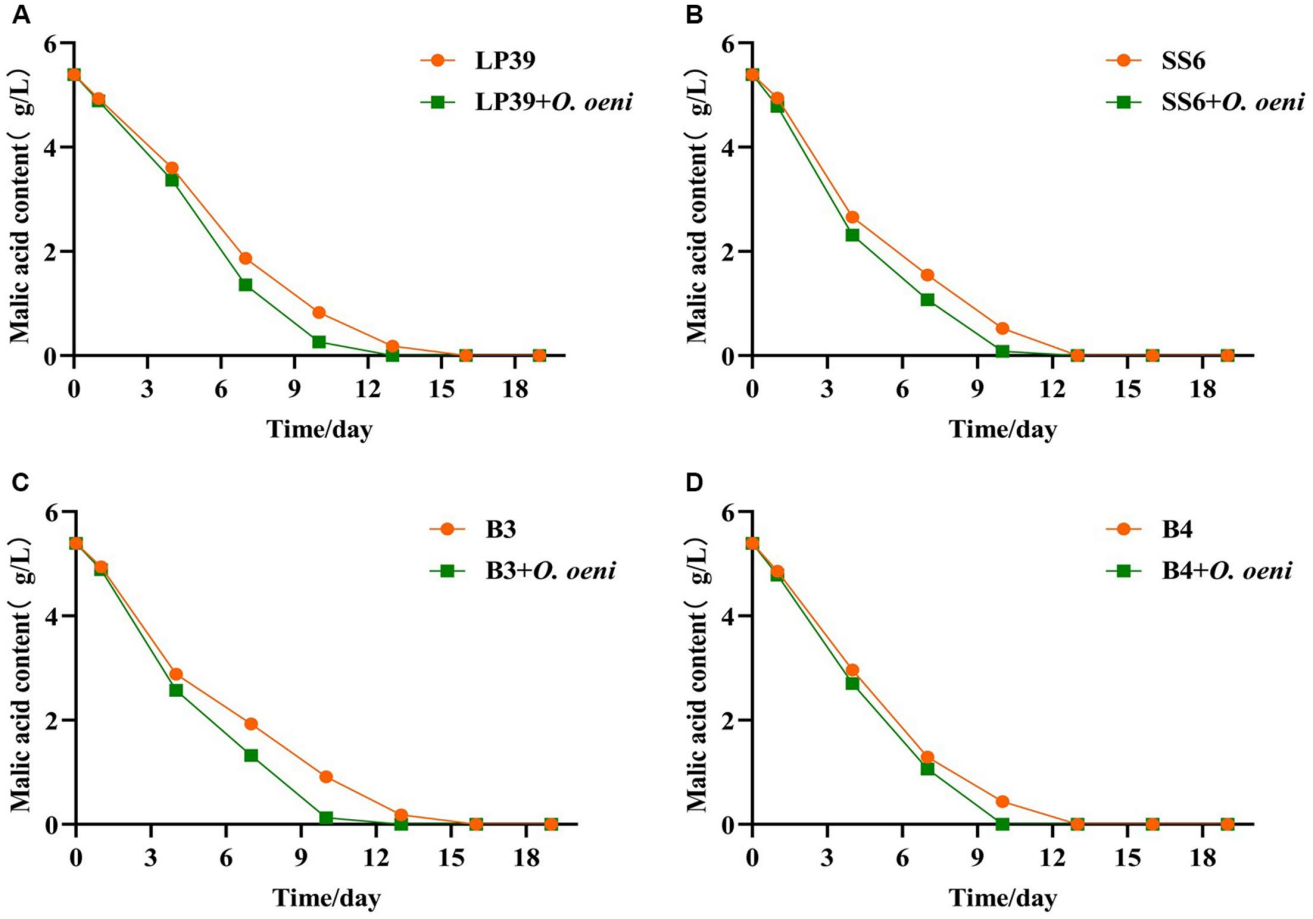
Changes in malic acid content in wine during fermentation with four strains of *L. plantarum*. LP39 **(A)**, SS6 **(B)**, B3 **(C)**, B4 **(D)**.

From the changes in malic acid during fermentation (Figure 5), and the changed in malic acid before and after malolactic fermentation with different strains were shown in Supplementary Table S2, the initial malic acid was 5.39 ± 0.03 g/L. With the inoculation of *L. plantarum*, the malic acid in all wine samples decreased significantly as fermentation progressed. And the malic acid consumption rate of the mixed strain fermentation samples was significantly faster than that of the single strain fermentation samples, and the malolactic fermentation was completed on the 10^th^ day (malic acid content <0.20 g/L) for all mixed strain fermentation samples and on the 13th day (malic acid content <0.20 g/L) for all single strain fermentation samples, indicating that *L. plantarum* can perform malolactic fermentation alone. The results of the plate counts were highly compatible with the PMA-CELL-qPCR method throughout the fermentation process and can be used as an alternative to plate counts for the viability of *L. plantarum* during wine fermentation.

## 4. Conclusion

In conclusion, this study successfully developed and implemented a PMA-CELL-qPCR method to accurately quantify the microbial population of *L. plantarum* in wine and wine-related matrices. This novel assay circumvented the need for conventional DNA extraction procedures and demonstrated the ability to discriminate between viable and non-viable microbial cells. Remarkably, the PMA-CELL-qPCR approach exhibited consistent microbial population counts when compared to the traditional plate counting assay typically employed in wine vinification processes, thereby offering substantial time and cost savings. Furthermore, the PMA-CELL-qPCR method facilitated rapid determination of microbial populations in wine samples without requiring sample dilution, while also ensuring specific measurements of target microorganisms.

## Data availability statement

The original contributions presented in the study are included in the article/Supplementary material, further inquiries can be directed to the corresponding author.

## Author contributions

JW and BW: conceptualization, software, formal analysis, and writing-original draft. ZC: methodology, investigation, and writing-review and editing. YC: formal analysis and software. SL: data curation and validation. BoZ: resources, supervision, and project administration. BaZ and DY: resources, supervision, writing-review, and editing. All authors contributed to the article and approved the submitted version.

## Funding

This work was financially supported by the Guangxi Science and Technology Base and Talent Special Project [Gui Ke AD21220159], Guangxi Academy of Agricultural Sciences Fundamental Research Project [Gui Nong Ke 2021YM12], and the Open Project Program of the College Student Innovation and Entrepreneurship Training Program from Beijing Forestry University (G202010022092 and G202010022093).

## Conflict of interest

The authors declare that the research was conducted in the absence of any commercial or financial relationships that could be construed as a potential conflict of interest.

## Publisher’s note

All claims expressed in this article are solely those of the authors and do not necessarily represent those of their affiliated organizations, or those of the publisher, the editors and the reviewers. Any product that may be evaluated in this article, or claim that may be made by its manufacturer, is not guaranteed or endorsed by the publisher.
