# Supplementary material for: A rapid and reliable method for the determination of Lactiplantibacillus plantarum during wine fermentation based on PMA-CELL-qPCR
Source: Front Microbiol. 2023 Jul 17;14:1154768. doi: 10.3389/fmicb.2023.1154768 (PMC10389660; doi:10.3389/fmicb.2023.1154768)
Supplement: Supplementary file 1 [file Data_Sheet_1.docx]

**Supplementary material**

Table S1. Physicochemical parameters of wines of two origins before and after fermentation

| Wine Origin | Reducing sugar (g/L) | | Malic acid (g/L) | |
| --- | --- | --- | --- | --- |
|  | AF Initial | End of AF | MLF Initial | End of MLF |
| Pinggu | 147.31 ± 2.56 | 1.58 ± 0.62 | 4.33 ± 0.54 | 0.18 ± 0.06 |
| Huailai | 142.64 ± 4.51 | 2.79 ± 0.7 | 3.97 ± 0.34 | 0.06 ± 0.02 |

Table S2. Different strains' changes in malic acid during malolactic fermentation

| Starins | Malic acid (g/L) | |
| --- | --- | --- |
|  | MLF Initial | End of MLF |
| LP39 | 5.39 ± 0.03 | 0.18 ± 0.03 |
| LP39+*O. oeni* | 5.39 ± 0.03 | 0.00 ± 0.00 |
| SS6 | 5.39 ± 0.03 | 0.09 ± 0.03 |
| SS6+*O. oeni* | 5.39 ± 0.03 | 0.00 ± 0.00 |
| B3 | 5.39 ± 0.03 | 0.18 ± 0.03 |
| B3+*O. oeni* | 5.39 ± 0.03 | 0.01 ± 0.01 |
| B4 | 5.39 ± 0.03 | 0.00 ± 0.00 |
| B4+*O. oeni* | 5.39 ± 0.03 | 0.00 ± 0.00 |
